# Supplementary material for: Associations Between Maximal Passive Knee Extension and Sagittal Plane Kinematic Patterns in Children with Spastic Cerebral Palsy: A Longitudinal Study
Source: J Clin Med. 2025 Dec 3;14(23):8567. doi: 10.3390/jcm14238567 (PMC12693222; doi:10.3390/jcm14238567)
Supplement: Supplementary file 1 [file jcm-14-08567-s001.zip › jcm-3916835-supplementary.pdf]

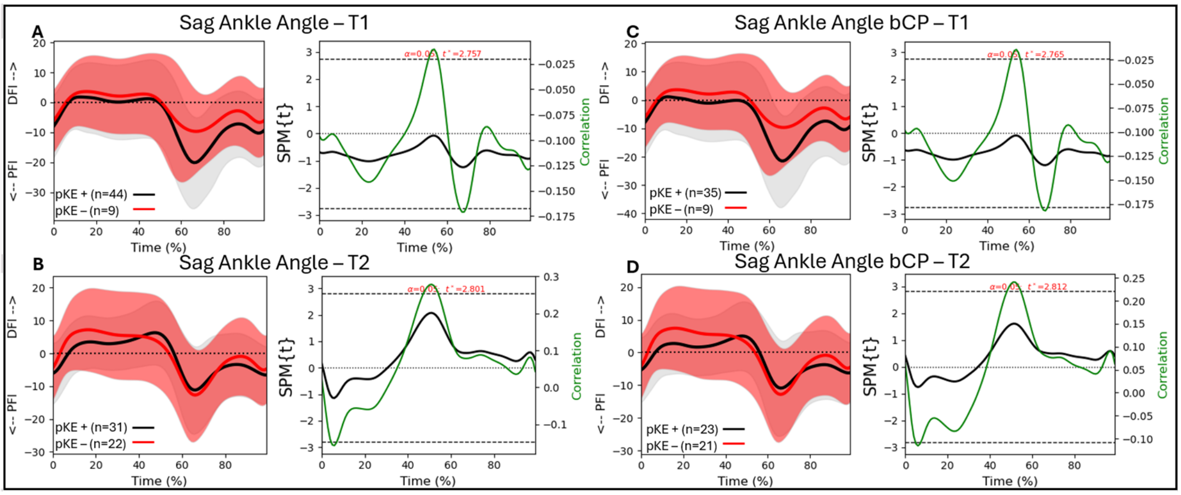

Figure S1: Association between sagittal ankle angle and passive knee range of motion. Sagittal ankle angle trajectories during a gait cycle derived from three-dimensional gait analysis for individuals with cerebral palsy and the associations with maximum passive knee extension (pKE) above (+) or below (-) a neutral position of the knee joint at clinical examination. The whole group of children with spastic CP (n=41) at A) timepoint T1 and B) timepoint T2, and the bCP group (n=22) at C) timepoint T1 and D) timepoint T2. PFl= plantarflexion; DFl = dorsiflexion.References.

Table S1. Intervention information on a group level for each patient. BCP = bilateral CP; UCP = unilateral CP; L = left; R = right; GMFCS = Gross Motor Function Classification level; BTX = botulinum neurotoxin A; IV = intervention.

Code	Diagnosis	GMFCS	INTERVENTION GROUP
**CP001**	BCP	I	NO IV
**CP002**	BCP	II	SURGERY
**CP003**	BCP	II	SURGERY
**CP004**	BCP	I	BTX
**CP005**	BCP	II	BTX + SURGERY
**CP006**	BCP	I	BTX
**CP007**	BCP	II	BTX + SURGERY
**CP008**	BCP	II	BTX
**CP009**	UCP R	I	SURGERY
**CP010**	BCP	I	SURGERY
**CP011**	BCP	I	BTX + SURGERY
**CP012**	UCP L	I	BTX
**CP013**	UCP L	I	BTX
**CP014**	UCP L	I	SURGERY
**CP015**	UCP R	II	SURGERY
**CP016**	UCP R	I	BTX + SURGERY
**CP017**	BCP	II	BTX
**CP018**	BCP	III	SURGERY
**CP019**	BCP	II	SURGERY
**CP020**	BCP	II	BTX + SURGERY
**CP021**	BCP	II	SURGERY
**CP022**	BCP	II	BTX
**CP023**	BCP	III	BTX + SURGERY
**CP024**	BCP	II	BTX
**CP025**	BCP	III	BTX
**CP026**	UCP L	I	NO IV
**CP027**	BCP	II	BTX
**CP028**	BCP	II	BTX + SURGERY
**CP029**	UCP L	I	BTX + SURGERY
**CP030**	BCP	I	SURGERY
**CP031**	UCP R	I	NO IV
